# Surveillance of antimicrobial resistance in veterinary medicine in the United States: Current efforts, challenges, and opportunities

**DOI:** 10.3389/fvets.2022.1068406

**Published:** 2022-12-20

**Authors:** Juliana M. Ruzante, Beth Harris, Paul Plummer, Raissa R. Raineri, John Dustin Loy, Megan Jacob, Orhan Sahin, Amanda J. Kreuder

**Affiliations:** ^1^Center for Environmental Health Risk and Sustainability, RTI International, Durham, NC, United States; ^2^National Animal Health Laboratory Network, National Veterinary Services Laboratories, U. S. Department of Agriculture, Animal and Plant Health Inspection Service, Ames, IA, United States; ^3^Department of Veterinary Diagnostic and Production Animal Medicine, College of Veterinary Medicine, Iowa State University, Ames, IA, United States; ^4^National Institute of Antimicrobial Resistance Research and Education, Ames, IA, United States; ^5^Department of Veterinary Microbiology and Preventive Medicine, College of Veterinary Medicine, Iowa State University, Ames, IA, United States; ^6^Nebraska Veterinary Diagnostic Center, School of Veterinary Medicine and Biomedical Sciences, University of Nebraska-Lincoln, Lincoln, NE, United States; ^7^Department of Population Health and Pathobiology, College of Veterinary Medicine, North Carolina State University, Raleigh, NC, United States

**Keywords:** surveillance, veterinary medicine, data sharing, antimicrobial stewardship, animal health, antimicrobial resistance (AMR)

## Abstract

Antimicrobial resistance (AMR) is a global problem facing human, animal, plant, and environmental health by threatening our ability to effectively treat bacterial infections with antimicrobials. In the United States, robust surveillance efforts exist to collect, analyze, and disseminate AMR data in human health care settings. These tools enable the development of effective infection control methods, the detection of trends, and provide the evidence needed to guide stewardship efforts to reduce the potential for emergence and further spread of AMR. However, in veterinary medicine, there are currently no known equivalent tools. This paper reviews efforts in the United States related to surveillance of AMR in veterinary medicine and discusses the challenges and opportunities of using data from veterinary diagnostic laboratories to build a comprehensive AMR surveillance program that will support stewardship efforts and help control AMR in both humans and animals.

## 1. Introduction

It is estimated that globally in 2019, almost 5 million human deaths were associated with bacterial antimicrobial resistance (AMR), including over 1.25 million deaths directly attributable to bacterial AMR (1). This places the global health impacts of AMR on par with HIV/AIDS and malaria (1). In the United States, conservative estimates indicate that annually more than 2.8 million people develop antibiotic-resistant infections and over 35,000 of those infected die (2). Furthermore, the American Veterinary Medical Association Committee on Antimicrobials has identified increasing numbers of infections associated with bacteria resistant to first-line antimicrobials in selected animal species in the United States, representing a challenge for veterinarians (3). From the One Health perspective, water, soil, and wildlife represent important AMR reservoirs contributing to the spread of resistance (4). Although AMR found in bacteria may occur naturally, the overuse of antimicrobials in humans, animals, and plants is accelerating the rapid spread of resistant bacteria and their genes between people, animals, and the environment (2, 5). Further, the COVID-19 pandemic slowed progress in addressing AMR and the incidence of some types of resistant bacteria significantly increased (6).

To address AMR, the United Nations Quadripartite (a collaborative framework of the Food and Agriculture Organization of the United Nations, United Nations Environment Programme, World Organization for Animal Health, and World Health Organization), the U.S. National Academies of Science, and the U.S. Centers for Disease Control and Prevention (CDC) promote a One Health approach of collaboration across human, animal, and environmental health sectors (2, 7–9). Surveillance and antimicrobial stewardship are among the core activities in this effort (2, 7, 8).

In the United States, centralized platforms regularly aggregate, analyze, monitor, and share AMR data collected from human healthcare facilities (10, 11). This enables the development of prevention and control measures and informs prescribing practices in health care settings. In animal health, there are currently no known equivalent tools. This paper presents the existing efforts in the United States that gather AMR data in animals and discusses the challenges and opportunities to build a comprehensive surveillance program for AMR in veterinary medicine using existing data from veterinary diagnostic laboratories (VDLs).

## 2. Current efforts

As a result of the two consecutive National Action Plans for Combating Antibiotic-Resistant Bacteria (CARB) (12, 13), the U.S. Food and Drug Administration (FDA), the U.S Department of Agriculture (USDA), and the CDC have made significant progress to improve the collection, analysis, and dissemination of AMR data from livestock and companion animals.

The *National Antimicrobial Resistance Monitoring System (NARMS)* is a collaboration between CDC, FDA, and USDA to monitor AMR among commensal bacteria (*Escherichia coli* and *Enterococcus*) and select pathogens (*Campylobacter, Salmonella, E. coli* O157) isolated from human patients, retail meats, cecal contents, and meat/poultry products collected at inspected slaughter and processing establishments (14). In partnership with NARMS, FDA's Veterinary Laboratory Investigation and Response Network (Vet-LIRN) and USDA's National Animal Health Laboratory Network (NAHLN) have additional monitoring programs for select pathogens from sick animals. As part of this, data for companion animals are combined and reported jointly through FDA's NARMS website (15). Standard methodology is used to obtain samples, conduct and interpret antimicrobial susceptibility testing (AST), and sequence the genomes (16). NARMS's strength is the ability to serve as a One Health platform for AMR and integrate data (phenotype and genotype) from several sources within the program. The data are shared in several ways, including through “NARMS Now: Integrated Data” (17), which is likely the most relevant tool for veterinary medicine as it allows users to access susceptibility results *via* an interactive format. However, to compare the different sources, NARMS uses human breakpoints to interpret the minimum inhibitory concentration (MIC) of all isolates to determine resistance (18, 19); this approach meets NARMS's goals but limits the immediate use of these data to support antimicrobial decision making in veterinary medicine as human clinical breakpoints often differ substantially from those in animal species (19, 20). The MICs and source data can be downloaded but are not readily available *via* the dashboard. NARMS Now provides genotypic resistance results for the present year, but phenotypic MIC data lag and the latest available is from 2019 (21).

For isolates sequenced by NARMS, the whole genome sequences (WGS) are frequently uploaded along with selected metadata into the National Center for Biotechnology Information (NCBI) database. NARMS has also developed Resistome Tracker (22), a global tool to explore resistance and other microbial features from a wide range of bacteria submitted to the NCBI (23). Resistome Tracker has current data and lets users explore and compare the distribution of different AMR genes by source (animals, environmental, etc.), country, and antimicrobial drug. There are no phenotype data on Resistome Tracker, thus its relevance in directly assisting veterinarians with stewardship efforts is unclear.

The *National Database of Antibiotic Resistant Organisms (NDARO)* is a cross-agency centralized database curated by NCBI. It provides access to AMR data to facilitate real-time surveillance of pathogenic organisms (23, 24). It is currently published as a beta version, but the Pathogen Detection Isolates Browser contains over 50 bacterial species from several sources including animals (24). Submissions come from across the United States including governmental and non-governmental institutions as well from around the world. NDARO's strength is the ability to aggregate and analyze WGS from a large number of organisms across the world. However, the use of this database in animal health is currently limited since the majority of the data comes from humans. Further, the metadata lacks the standardization necessary (e.g., isolate source, host terms, and geospatial data are not consistently used) to allow robust searches to be conducted within and across animal species. Phenotype data (i.e., AST) are only available for a small portion of isolates (24). The user interface is geared toward researchers, making it difficult for veterinarians to consult for aiding in clinical decision making or surveillance use (24).

The *Veterinary Laboratory Investigation and Response Network (Vet-LIRN)* is a collaboration between FDA and VDLs to promote human and animal health. As a part of the CARB plan, Vet-LIRN was tasked to develop, expand, and maintain AST and WGS testing of selected veterinary pathogens (*E. coli* and *Staphylococcus pseudintermedius* from dogs, and *Salmonella enterica* from any animal species) isolated at VDLs (25). Vet-LIRN's strength is the ability to obtain both WGS and AST results from selected isolates. Results from 2018 and 2019 isolates from sick dogs are available *via* a dashboard hosted in combination with NARMS and NAHLN that allows users to visualize the isolates by organism and state (15). For *E. coli* and *S. pseudintermedius*, users can explore the percentage resistance with both gene and MIC distributions (15). Whenever available, veterinary breakpoints are used to interpret resistance, and samples are classified as either urinary tract infections (UTI) or non-UTI specimens to enable interpretation (15). While grouping specimens into UTI and non-UTI categories may be considered appropriate from a laboratory perspective (19, 20), further categorization by specimen type could be useful to veterinary practitioners to aid clinical decisions and resistance interpretation.

The *National Animal Health Laboratory Network (NAHLN)* started as a pilot in 2018. It was created by USDA Animal and Plant Health Inspection Service (APHIS) to demonstrate the viability of implementing a sampling stream for monitoring AMR profiles in animal pathogens routinely isolated by U.S. VDLs. The project collects and aggregates AMR profiles for livestock and companion animals that are clinically ill, and all VDLs that submit their data use standardized methods and AST panels (26). VDLs submit AST data for a specific number of isolates per year and only for *E. coli* from cattle, swine, poultry, horses, dogs, and cats; *Salmonella enterica* from cattle; *Mannheimia haemolytica* from cattle; *Streptococcus suis* from swine, *Pasteurella multocida* from chicken and turkeys; *Streptococcus equi* subsp. *equi* and *S. equi* subsp. *zooepidemicus* from horses; and *Staphylococcus pseudintermedius* from dogs and cats. The strength of NAHLN is the ability to use existing VDL data and quickly disseminate information on these pathogen-animal combinations. An online dashboard on NAHLN's website enables the visualization of MIC distributions by selecting the pathogen, species, drug, and date under “MIC Table” (26). When available, veterinary breakpoints are used for interpretations (26, 27). For dogs and cats, data from UTIs are displayed separately from all the other specimens. Additionally, data from companion animals are combined with Vet-LIRN data and reported jointly through FDA's NARMS website (15). With the increased breadth of organisms, the use of veterinary breakpoints and the up-to-date information, NAHLN provides the type of data needed to improve stewardship in veterinary medicine, however, results are presented only at the national level and at this time the number of pathogens and isolates remains limited.

The *National Animal Health Monitoring System (NAHMS)*, administered by USDA APHIS, performs national studies on health and management practices of domestic livestock, including poultry, populations on a rotating schedule (28). Participation by producers is voluntary, with participants selected using a weighted statistical approach to provide national estimates. Many studies involve the collection of biological samples that are examined for a range of issues depending on the goal of the study (29–31). The recent NAHMS Health Management study on U.S. Feedlots 2021 included questions about antimicrobial stewardship (32). The Swine 2021 study also included AST on fecal cultures from grower/finisher pigs for *Salmonella, E. coli, Campylobacter*, and *Enterococcus* (33). NAHMS's strength is the ability to collect data from healthy animals *via* a systematic approach. Results can be found in reports and dashboards publicly available on APHIS's website (34). The NAHMS reports provide country-level AMR results (e.g., number and percentage of isolates resistant by antimicrobial agent), but more details (e.g., MIC distribution) can be found in specific peer-reviewed publications (35–38). While data are not collected annually from each commodity group, AMR results can be compared with those from previous years to assess trends over time in healthy animals.

*Other efforts*, including additional regional and national pilots, exist to collect AMR data from animals. The main strength of these additional efforts is the ability to explore new formats of data collection, aggregation, and dissemination, but to date results of these efforts have not been made public (39–42).

## 3. Challenges and opportunities

Despite the growth and expansion of the existing AMR surveillance systems to collect relevant data for veterinary medicine, the currently available programs remain heavily focused on zoonotic pathogens and are limited to few organisms of animal health relevance. The animal data available are often aggregated at the national (e.g., NAHMS, NAHLN) or state level (i.e., NARMS/Vet-LIRN). Except for WGS that are frequently uploaded to NCBI, NAHLN, and NARMS genotype resistance results, data availability is often delayed by several years. These factors make the current systems insufficient to provide researchers, commodity groups, and veterinary practitioners with current and relevant evidence needed to develop control measures and guide stewardship practices, highlighting the need for a comprehensive, real-time surveillance program for AMR in veterinary medicine.

Central to the development of such efforts is the ongoing and timely collection of AMR data. However, surveillance activities designed to generate these types of information are complex, expensive, and time consuming to implement; therefore, leveraging and combining existing datasets can be valuable. Unlike human medicine where collection of these data is incentivized by requirements for Medicare reimbursement, state regulations, or other health system subsidies, veterinary medicine has few mechanisms to incentivize generating or sharing this type of data (43).

The model piloted by NAHLN to aggregate VDL data represents a unique opportunity. VDLs routinely receive and test an array of clinical samples from diverse animal species for AST. Many perform MALDI-TOF mass spectrometry for bacterial identification, and some may perform WGS for a limited number of pathogens. Although biased against healthy animals, VDLs provide a rich source of data for AMR surveillance in diseased animals. The ability to aggregate, analyze, and share this type of information in a manner that is region specific and timely would allow for (i) developing cumulative susceptibility information to guide prescribing practices, (ii) monitoring resistance trends, (iii) detecting emerging diseases, and (iv) potentially improving clinical decision making [i.e., generation of new breakpoints and epidemiological cut-off values (ECVs or ECOFFs)]. However, using VDL data to build a centralized surveillance system has many challenges which we have summarized below:

1. *Harmonization of AST methods*. Many VDLs rely on AST methodology and quality standards control recommended by the Clinical and Laboratory Standards Institute (CLSI) (44). However, many techniques, including disc diffusion assays, broth microdilution, and epsilometer testing (E test) are available. This can lead to inconsistent results between labs, impacting data quality and limiting comparisons. While many VDLs conduct their own quality control and method validation, NAHLN has recently incorporated annual proficiency testing as part of their procedures to help improve future methods harmonization.
2. *Standardized nomenclature and ontology*. Currently, veterinarians submitting data and VDLs use a range of terms and abbreviations that are not consistent across institutions. Harmonization of terminology is critical for aggregating and analyzing data across different sources and for developing a data ecosystem based on the FAIR principles of research (i.e., findable, accessible, interoperable, and reusable) (45). While ontology platforms such as the Systematized Nomenclature of Medicine (SNOMED) exist, the need for further refinement of these platforms related to veterinary AST remains, and the ontology must be more widely adopted across labs to be effective.
3. *Standardized data interpretation of resistance*. In veterinary medicine, there is a severe paucity of established clinical breakpoints. Furthermore, human and veterinary CLSI standards used to interpret AST results are frequently updated. Therefore, reporting interpretative criteria (i.e., susceptible, intermediate, or resistant) may differ across laboratories over time. While diagnostic interpretations are often easier to collect and summarize in the short term, this approach compromises long-term accuracy of the dataset. To ensure resistance is being determined consistently across the years using the latest CLSI standards, VDLs need to provide raw AST data such as MICs or zone diameters to surveillance programs rather than interpretations. Any reliable analysis of temporal trends will need consistency in the interpretation of MIC or zone diameter data. MIC data also allow for the use of both human and veterinary breakpoints and for the determination of ECVs, which are helpful in the absence of clinical breakpoints and for One Health comparisons (46).
4. *Standardized data reporting*. VDLs produce large volumes of data and their IT infrastructure is typically lab-specific and designed to produce reports back to their clients rather than to surveillance programs. Further, AST results are not always automatically linked to the laboratory information management systems (LIMS) that contain the metadata needed to interpret it. LIMS are also not set up to easily format, standardize, export, and transfer this information on an ongoing basis. Data sharing thus represents a significant burden to VDLs and might prevent their participation in surveillance programs that require specific formats and metadata. Data submitted to NAHLN and Vet-LIRN, for instance, must use standardized diagnostic methods and be in a certain format. This requires significant human labor and data manipulation, in addition to specialized equipment and vendors. Ongoing advances in IT and programming are assisting in making the automatization of such tasks possible, but this remains a significant hurdle for many smaller labs. Thus, the development of a robust IT infrastructure that can handle high volumes of data in a continuous manner and remove the burden from VDLs by accepting multiple formats is critical for the development of a national centralized AMR surveillance program.
5. *Representativeness*. Most VDL samples come from sick animals and often represent the most severe cases and those that failed treatment, and thus may not be representative of the general population. While this is also the case in human medicine surveillance (10, 11), the difference is that ASTs are performed less frequently in veterinary medicine due to associated costs (47). AST test design for clinical samples is also focused around known breakpoints, and thus the MIC data range that is generated may be very limited leading to challenges in interpretations and comparisons across a One Health spectrum. Thus, VDL AST data are not ideal for all applications (i.e., development of antibiograms for first-line empirical therapy). These limitations, however, do not discredit the value of utilizing VDL AST data in a centralized national surveillance program. For example, several European countries have developed AMR surveillance systems for diseased animals (47), and the European Union has an ongoing effort to develop a standardized and continuous program at the EU level modeled after the European Antimicrobial Resistance Surveillance Network in humans (48, 49).
6. *Data accuracy*. Much of the metadata associated with VDL data are self-reported by veterinarians, owners, or producers. Thus, there is an inherent risk for inaccuracy of the data as it relies upon the effort applied by the submitter to include all relevant and accurate information. In addition, many livestock production systems in the U.S. are large, multi-site and/or state operations. Therefore, if geographic localization of the source of bacterial isolates is the goal, it is important to ensure that the actual site of isolation (not billing address) is utilized. Some VDLs incentivize inclusion of premise identification numbers, which could assist in this process; however, use of these data would require balancing the data protection needs of stakeholders.
7. *Data confidentiality and security*. Accredited VDLs have a requirement to protect their clients' data, many of whom might be concerned by Freedom of Information Act (FOIA) requests, potential misuse and misinterpretation of information, and the risk of reidentification. To develop an effective AMR surveillance system in veterinary medicine, the needs and concerns of the different data users and those providing data must be balanced. For example, the appropriate geospatial level of definition that can protect confidentiality while providing useful information needs to be carefully defined, so no individual operation or animal population can be identified. Models for data security and confidentiality are well-described and widely used in dealing with sensitive information such as personal identifiable information and should be explored and adapted to protect AMR data from animal sources. Without these protections, stakeholders may be unwilling to participate in surveillance efforts.
8. *Relationship between AMR and antimicrobial use (AMU)*. AMU data from animals in the United States are often not connected with AMR data (50–55), however it is critical to evaluate their relationship with the development of resistance. While most VDLs request AMU information as part of the clinical history, the provision of these data are intermittent at best and not systematically collected to allow for associations with AMR results. Models for improving the collection of AMU in the VDL submission process should be explored in the future.

## 4. Conclusion

Challenges and limitations exist, but there is a unique opportunity to learn from existing initiatives and leverage the rich dataset from VDLs using state-of-the art technology to build an ongoing, real-time, and geo-specific AMR surveillance system for animal health in the United States. The data generated could be used to provide evidence-based clinical practices that can support and monitor successful prevention, control, and stewardship initiatives in veterinary medicine. Further, if enough high-quality data and extended dilution AST results become available, it could inform the development of new breakpoints in veterinary medicine and ECVs for One Health comparisons, plus provide much needed data regarding less common pathogens in major animal species and common pathogens of minor animal species, such as sheep and goats.

The integration of veterinary AST data with human, plant, water, and other environmental data, as well as with AMU data, were not discussed here, but are also imperative. Any effort to build a more comprehensive surveillance system to track AMR in animals must prevent redundancy, and ensure data integration across existing platforms. Also not discussed here and critical for the success of any surveillance system is sustained funding; prioritization of federal resources dedicated to AMR surveillance in animals is critical for the development of the IT framework, the standardization efforts, and to incentivize VDLs to participate. Finally, AMR is a global One Health issue; efforts developed in the United States should also consider the long-term goal of providing the ability to track AMR globally in both human and animal populations.

## Author's note

The authors declare that they have been involved in efforts to develop pilots for a centralized data coordinating center in veterinary medicine. The work gave them the inspiration for this article.

## Author contributions

All authors listed have made a substantial, direct, and intellectual contribution to the work and approved it for publication.
